# Health risk assessment of selected metals through tap water consumption in Upper Silesia, Poland

**DOI:** 10.1007/s40201-020-00579-5

**Published:** 2020-10-31

**Authors:** Rajmund Michalski, Paulina Pecyna-Utylska, Joanna Kernert, Katarzyna Grygoyć, Justyna Klyta

**Affiliations:** Institute of Environmental Engineering, Polish Academy of Sciences, Zabrze, Poland

**Keywords:** Tap water, Metals, Health risk assessment, Upper Silesia, Poland

## Abstract

The research focused on assessing the risk to human health resulting from the content of selected Cr, Co, Mn, Cu, Ni, Pb, As, Zn and Sr metals in tap water supplied by Upper Silesia Water Plant to the inhabitants of the Upper Silesia region (Poland). It is the main supplier of drinking water to several million inhabitants of this agglomeration. Samples were taken and analyzed quarterly in 2019. The sampling points were chosen to help identify the source when an elevated level of a particular contaminant is observed. ICP-MS and ICP-OES have been used to measure the concentrations of those elements. The chronic daily intake (CDI), hazard quotient (HQ) and hazard index (HI) results for non-carcinogenic risk assessment of metals in tap water has been assessed. CDI values of non-carcinogenic metals were higher in children than in adults; the CDI values for adults and children were found in the order of: Zn > Sr > Cu > Mn > Ni > Pb > Cr > Co > As. All the studied metals had HQ values below 1, the risks caused by the non-carcinogenic metals decreased in the following order: Zn > Cu > Co > As > Sr > Pb > Cr > Ni > Mn. HI values were also less than 1, that meaning that the analyzed tap water is safe for human consumption. The concentration of As, Cr, Cu, Mn and Ni in analyzed tap water is in accordance with Polish and international requirements.

## Introduction

Assessment of the human health risk comprises qualitative and quantitative evaluation of the human’s exposure to contamination originating mainly from environment and food, in particular in drinking water. The quality of water consumed has a strongly impact on our health and well-being. Human life strongly depends on the quality drinking water intake required to prevent any risks to human health. The consumption of water containing a certain amount of some metals may lead to health problems such as cancer in humans [1, 2]. Some metals and metalloids such as zinc, iron, selenium cobalt, copper, chromium, vanadium, or molybdenum are essential elements for the growth and reproduction, but their accumulation in excess in the human body is undesirable. In turn, non-essential metals such as lead and cadmium having no positive role in the metabolic activities can cause toxic effects on the body tissues [3]. Thus, it is extremely important to obtain reliable results in all laboratories involved in drinking water analysis [4].

Metals and metalloids have been determined with classic and instrumental methods. Classic methods are usually labor-intensive, require toxic chemicals, and often they do not provide full automation. When spectrometric methods appeared, the classic metal determination methods started to lose their significance [5]. At present, they have been nearly completely replaced with the instrumental methods including hyphenated techniques [6, 7].

The quality of drinking water is regulated at national and international levels [8, 9].In Poland it is Regulation of the Minister of Health from December 7th, 2017 [10]. Limit values for selected metals and metalloids as well as pH and conductivity recommended by WHO, US EPA and Polish regulation are given in Table 1. Tests for the quality of drinking water are carried out by specialized laboratories in accordance with the requirements described in the relevant regulations. Nevertheless, consumers are sometimes dissatisfied with the quality of tap water, primarily because of its taste, color or smell. In addition, they indicate that the composition of water in the water plants may be different than in the tap water, mainly because of water transfer systems [11]. It may results from sedimentation of the some contaminants, especially metals by corroding the water pipes and the washing-up through the water distribution system pollutes the tap water. The used materials in the household piping are transferred to the drinking water through the contacting corrosive water with pipes, valves, fittings, as well as municipal and domestic distribution networks. Due to the importance of this issue, several studies have been conducted to investigate the chronic health effects of exposure to metals from drinking water consumption worldwide [12, 13].

**Table 1:** Limit values for selected parameters in drinking water

Parameter	WHO [8]	US EPA [9]	Polish regulation [10]
Cr (total), mg/L	0.05	0.1	0.05
Co, mg/L	–	–	**–**
Mn, mg/L	0.4	–	0.05
Cu, mg/L	2.0	1.3	2.0
Ni, mg/L	0.07	–	0.02
Pb, mg/L	0.01	0.015	0.01
As, mg/L	0.01	0.01	0.01
Zn, mg/L	–	–	**–**
Sr, mg/L	–	–	**–**
pH	6.5–8.5	6.5–8.5	6.5–9.5
Conductivity, μS/cm	–	2500	2500

In Poland around 8700 plants producing drinking water exists, of which over 90% of them small are water plants, producing up to 1000 m^3^ of water per day. The largest of them - Upper Silesia Water Plant - is located in Katowice and supplies drinking water to several million inhabitants of this agglomeration. The population density is the highest in Poland and one of the highest in Europe, negatively contributing to the irreversible changes in the environment. It is a highly developing industrial region, with many hard coal mines, cookeries, heat and power plants, and steel works – all of these having a negative impact on the environment, including the quality of surface and groundwater [14, 15].

The aim of the study was to assess the content of selected metals (Cr, Zn, Co, Mn, Cu, Ni, Pb, As, Sr, and Zn) in drinking water collected at 18 points (households) in the Upper Silesia Voivodeship and assessment of health risk for its consumers. To the best of our knowledge, such research in this area has not yet been carried out.

## Materials and methods

### Sampling

**Fig. 1:**
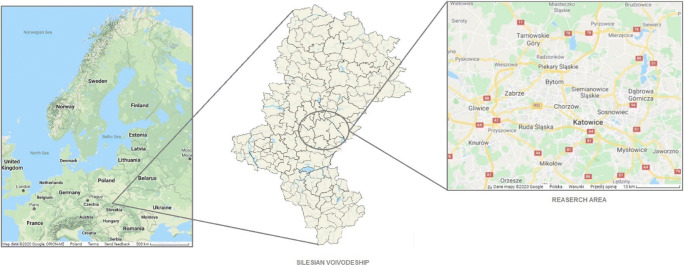
Study area and sampling points [16–18]

Tap water samples were taken quarterly from 18 selected locations in 8 cities (Zabrze, Bytom, Ruda Śląska, Piekary Śląskie, Katowice, Radzionków, Sosnowiec, Mysłowice) in Silesian Voivodeship, Poland (Fig. 1. and Table 2).

**Table 2:** Sampling points location in alphabetical order

1	Bytom, Estreichera St.	10	Radzionków, Adamieckiego St.
2	Bytom, Felińskiego St.	11	Ruda Śląska, Kingi St.
3	Bytom, Modrzewskiego St.	12	Ruda Śląska, Ogrodowa St.
4	Bytom, Podhalańska St.	13	Sosnowiec, Orląt Lwowskich St.
5	Katowice, Cicha St.	14	Zabrze, 3-th Maja St.
6	Katowice, Marcina St.	15	Zabrze, Damrota St.
7	Mysłowice-Wesoła, Norwida St.	16	Zabrze, Sikorskiego St.
8	Piekary Śląskie, Jana Ludygi St.	17	Zabrze, Tuwima St.
9	Piekary Śląskie, Ogrodowa St.	18	Zabrze, Tyska St.

Total 72 tap water samples were collected quarterly in 2019 in resident’s flats, in the kitchens. The water supplier for these consumers is Upper Silesia Water Plant. The raw water comes from surface water intakes, the composition of which varies depending on the seasons. That is why the research was conducted at any time of the year (spring, summer, autumn and winter). When sampling, the tap was turned on for 2 min prior to water collection in order to discharge the residual water deposition in the pipeline; each sampling was performed in triplicate. The samples were collected in pre-cleaned HDPE bottles and transported to the laboratory after acidification with concentrated ultrapure HNO_3_ to pH <2. Before acidification, the pH and conductivity were determined. All glassware and bottles used in the study were washed and rinsed with doubly distilled water. All chemicals used were of analytical grade, and the doubly deionized water was produced by Milli-Q-Gradient ultra-pure deionized water (Milli-Q-Gradient ZMQ5V001, Millipore). Tap water samples were filtrated through 0.22 μm PES syringe filters before analysis.

### Reagents

Standard solutions were obtained by appropriate dilution of a multi-standard metals Mix 1 (Sigma-Aldrich, Switzerland), using Milli-Q-Gradient ultra-pure deionized water (Milli-Q-Gradient ZMQ5V001, Millipore), which conductivity was <0.05 μS/cm. The Merck suprapure (Germany) 65% nitric acid was used for acidifying calibration solutions and samples. All standards and blank were prepared daily by using analytical balance (PX224M/1, Pioneer), directly before sample analysis. The calibration blank consisted of the acid solution was used to prepare the standards.

In order to calibrate the pH-meter, five standard solutions (Reagecon, Ireland) with nominal values: 1.675, 4.0, 6.8, 9.225 and 12.0 were used. Two standard solutions (Reagecon, Ireland) with nominal values: 84 μS/cm and 12,880 μS/cm were used for calibration the conductometer.

### Apparatus

pH and conductivity were determined using an InoLab IDS Multi 9310 device equipped with a pH-metric electrode IDT SenTix®940 and a conductometric sensor TetraCon 925 IDS (both from WTW, Poland) according to EN ISO 10523:2012 and EN 27888:1999 standards. The determinations of As, Co, Cr, Cu, Mn, Ni, Pb were carried out using a PerkinElmer inductively coupled plasma mass spectrometer ICP-MS Elan 6100 DRC-e (Shelton, USA). The operating conditions are: radio frequency power 1125 W; plasma gas flow 15 L/min; auxiliary gas flow 0.9 L/min; lens voltage 6.5 V. Nebulizer gas flow was optimized as needed for highest signal in the range of 0.78–0.82 L/min. The instrument effectiveness was checked daily before measuring samples by measuring an optimization solution (Elan 6100 Setup/Stab./Masscal) with a concentration 10 μg/L of appropriate elements (Mg, Cu, Rh, Cd, In, Ba, Ce, Pb, U) in 1% HNO_3_. In order to minimalize matrix effects, standards, blanks and samples were measured using ^103^Rh as internal standard. Solution of 10 μg/L Rh was added to all solutions and samples on line, by teeing in tubing on peristaltic pump.

The concentration of Zn and Sr were determined using ICP-OES inductively coupled optical emission spectrometry (Avio 200 - Perkin Elmer), according to ISO 11885:2009. The instrumental parameters were: plasma gas flow - 10 L/min; auxiliary gas flow - 0.2 L/min; nebulizer gas flow - 0.6 L/min; radio frequency power - 1400 W; pump flow - 1.0 mL/min.

All standards and blank were prepared daily by using analytical balance, directly before sample analysis. The calibration blank consisted of the acid solution was used to prepare the standards. The validation methodology for the analyzed metals was based on the standard addition method. The recovery was determined by measuring real samples to which a known analyte amount was added. Limits of detection (LOD) were calculated from calibration curves of analyzed metals (Table 3) and were based on the following relationship:

**Table 3:** LOD for selected metals

Metal	LOD, μg/L
Mn	0.20
Co	0.01
Ni	0.14
Pb	0.21
As	0.58
Cr	0.08
Cu	0.38
Sr	10
Zn	50

$$ \mathrm{LOD}=\frac{3.3\times {\mathrm{S}}_{\mathrm{d}}}{\mathrm{b}} $$

where: S_d_ - standard deviation value, b - the slope of the calibration curve.

### Health risk assessment

Health risk assessment as a result of exposure to the chemical substance is the process consisting of four stages: hazard identification, exposure assessment, dose–response assessment, and risk characterization. The basis for the exposure assessment is to determine the intake dose. The Chronic Daily Intake (CDI, mg/kg × day) was calculated using following equation:

19 $$ \mathrm{CDI}=\mathrm{C}\frac{\mathrm{CR}\times \mathrm{EF}\times \mathrm{ED}}{\mathrm{BW}\times \mathrm{AT}} $$

where: C – average concentration of metals at exposure, mg/L; CR – contact rate, L/day; EF – exposure frequency, days per year; ED – exposure duration, years; BW – body weight, kg; AT – period over which exposure is averaged, days.

In Table 4 the values of parameters for CDI calculation are reported.

**Table 4:** Human health risk assessment – the values of parameters for calculation [16, 19]

Parameter	Value		
	Non-carcinogenic risk assessment		Carcinogenic risk assessment
	Adult	Child	
CR, L/day	2	1	2
EF, days per year	365		
ED, years	30	6	70
BW, kg	70	15	70
AT, days	ED × 365		

Non-carcinogenic health risk assessment is based on the Hazard Quotient (HQ), which was calculated using equation:

19 $$ \mathrm{HQ}=\frac{{\mathrm{CDI}}_{\mathrm{non}-\mathrm{carcinogenic}}}{\mathrm{RFD}} $$

where RFD is the Reference Dose Factor [mg/kg/day].

The Hazard Index (HI) is the sum of the HQ and is calculated when several metal(loid)s are studied; the critical value for HQ and HI is 1.

The individual excess lifetime cancer risk (IELCR) is calculated for carcinogenic substances using equation:

19 $$ \mathrm{IELCR}={\mathrm{CDI}}_{\mathrm{carcinogenic}}\times \mathrm{SF} $$

Where SF is Slope Factor (mg/kg/day).

Total IELCR is calculated when several carcinogenic metal(loid)s are studied. The critical value for IELCR and total IELCR is 10^−6^.

The RFD and CF values were acquired from: The Risk Assessment Information System, Provisional Peer-Reviewed Toxicity Values Assessments, US EPA Integrated Risk Information System, 2018 Edition of the Drinking Water Standards and Health Advisories Tables [26–28].

## Results and discussion

### Conductivity, pH, and metals concentration in analyzed tap water

The conductivity and pH range in tap water samples are given in Table 5; the measured values were compliant with limit values, recommended for drinking water by Polish regulation, WHO, and US EPA.

**Table 5:** Conductivity and pH in tap water samples

Parameter	Unit	Range [min-max]	Mean	Median	SD
Conductivity	μS/cm	157–794	404	456	158
pH	–	7.34–8.39	7.91	7.89	0.275

**Fig. 2:**
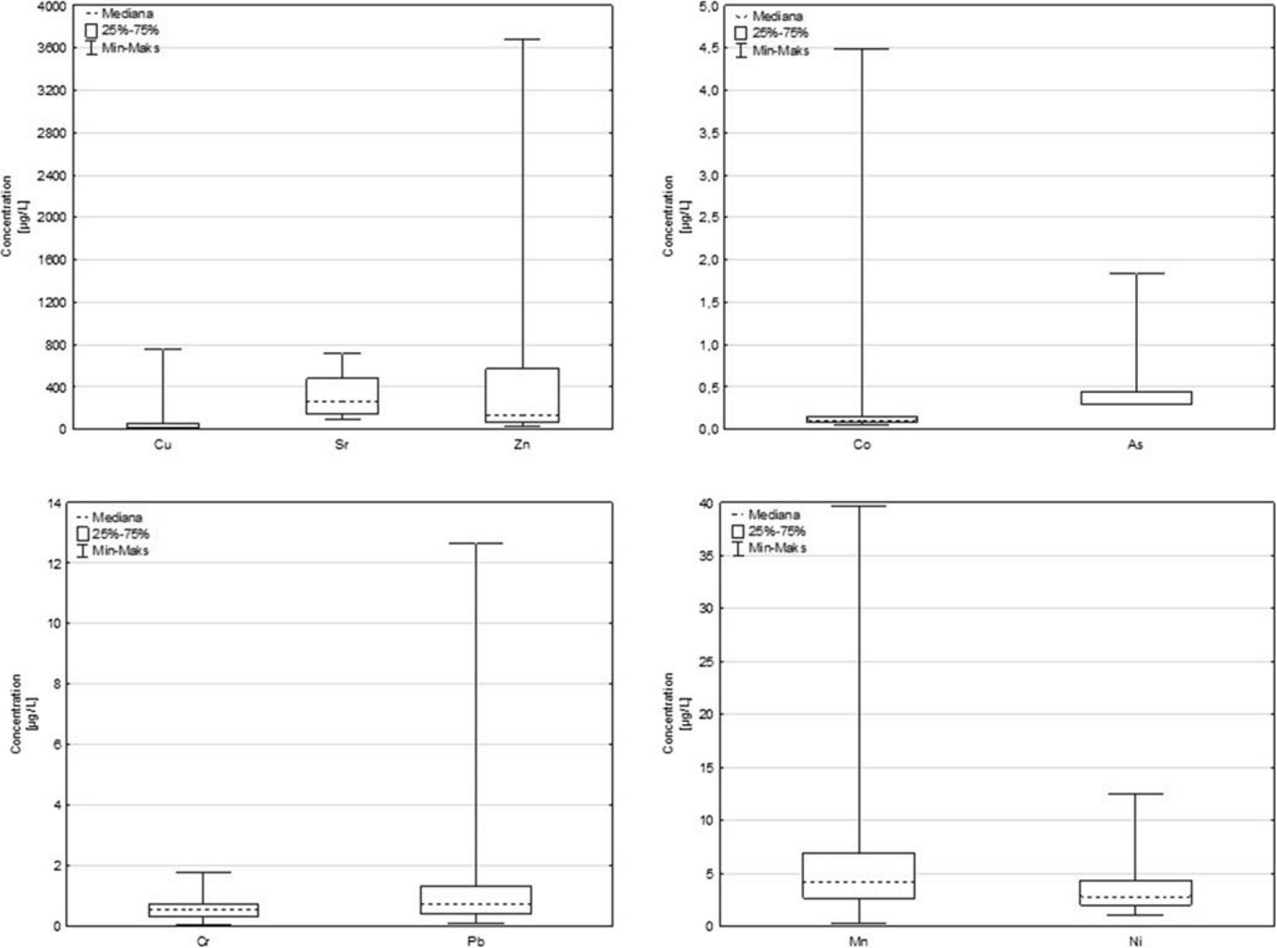
Minimal and maximal concentration, and median values for selected metals in tap water sample

Due to the very large number of results obtained (4 × 18 samples × 9 elements = 648 analyzes) on Fig. 2. are shown the minimum and maximum values and median values for all metals determined. For results below the detection limit, the half-LOD value method was used to create the graphs. The median concentration of selected metals in examined tap water, were as follow: Sr (260) > Zn(133) > Cu(23.1) > Mn(4.21) > Ni(2.85) > As(0.85) > Pb(0.75) > Cr(0.60) > Co(0.11), μg/L. The obtained results of individual metals concentrations present a wide range of values: As <0.58–1.84 μg/L, Co 2.71–4.50 μg/L, Cu 2.71–754 μg/L, Cr <0.08–1.78 μg/L, Mn 0.35–39.6 μg/L, Ni 1.06–12.6 μg/L, Pb <0.21–12.6 μg/L, Sr 89.0–720 μg/L. In addition 75% results for As was under LOD level. These broad concentration ranges for individual metals correspond to various sources of water supply for residents of individual cities and even parts of them. With regard to legal regulations (Table 1), it was found that the concentration of As, Cr, Cu, Mn and Ni in tap water meets the requirements. For Pb one of obtained values was over the WHO and Polish requirements: 12.64 μg/L. Moreover, there are no set limits for Co, Sr and Zn concentration in tap water.

### Health risk assessment

The CDI, HQ and HI results for non-carcinogenic risk assessment of metals in tap water are presented in Table 6. It was observed that the CDI values of non-carcinogenic metals were higher in children than in adults. The CDI values for adults and children were found in the order of: Zn > Sr > Cu > Mn > Ni > Pb > Cr > Co > As. The risks (HQ) caused by the non-carcinogenic metals decreased in the following order: Zn > Cu > Co > As > Sr > Pb > Cr > Ni > Mn. All the studied metals had HQ values below 1. Also HI values were less than 1. It means that the analyzed tap water is safe for human consumption.

**Table 6:** Non-carcinogenic risk of metal(loid)s in tap water

Metal	Concentration, mg/L	RFD, mg/kg/day	CDI, mg/kg/day		HQ	
			Adult	Child	Adult	Child
Cr (total)	5.98E-04	3.00E-03^*^	1.71E-05	3.98E-05	5.69E-03	1.33E-02
Co	3.13E-04	3.00E-04	8.95E-06	2.09E-05	2.98E-02	6.96E-02
Mn	5.46E-03	1.40E-01	1.56E-04	3.64E-04	1.11E-03	2.60E-03
Cu	5.67E-02	4.00E-02	1.62E-03	3.78E-03	4.05E-02	9.45E-02
Ni	3.67E-03	2.00E-02	1.05E-04	2.45E-04	5.24E-03	1.22E-02
Pb	1.34E-03	3.60E-03	3.84E-05	8.96E-05	1.07E-02	2.49E-02
As	2.52E-04	3.00E-04	7.19E-06	1.68E-05	2.40E-02	5.59E-02
Zn	5.00E-01	3.00E-01	1.43E-02	3.33E-02	4.76E-02	1.11E-01
Sr	3.05E-01	6.00E-01	8.71E-03	2.03E-02	1.45E-02	3.39E-02
HI = ∑HQ_i_					1.79E-01	4.18E-01

^*^RFD for chromium (VI) [29]

The CDI, IELCR and total IELCR results for carcinogenic risk assessment of metals in tap water are presented in Table 7. The CDI values were found in the order of: Pb > Cr > As. For carcinogenic substances, the acceptable threshold is 10^−6^. The risks caused by the carcinogenic metals decreased in the following order: As > Cr > Pb, and the individual excess lifetime cancer risks were higher than 10^−6^ for As (1.08E-05) and Cr (8.54E-06). In case of chromium high IELCR value is caused by lack of the slope factor (SF) for total chromium, so for calculation SF for chromium (VI) was used. Chromium speciation analysis would be required to determine the chromium (VI) concentration in tap water samples. It could be assumed that IELCR for chromium (VI) was below 10^−6^. IELCR for arsenium was higher than 10^−6^, but concentration of As in tap water samples was below the maximum value recommended by Polish regulation, WHO, and US EPA. Total IELCR was also higher than 10^−6^. The US EPA considers acceptable for regulatory purposes a cancer risk in the range of 10^−6^ to 10^−4^.

**Table 7:** Carcinogenic risk of metals in tap water

Metal	Concentration, mg/L	CDI, mg/kg/day	SF, mg/kg/day	IELCR, mg/kg × day
Cr (total)	5.98E-04	1.71E-05	5.00E-01^*^	8.54E-06
Pb	1.34E-03	3.84E-05	8.50E-03	3.27E-07
As	2.52E-04	7.19E-06	1.50E+00	1.08E-05
∑IELCR				1.96E-05

*SF for chromium (VI)

## Discussion

For example, Stegavik [20] tested the heavy metal pollution in the drinking water distribution network of Trondheim (Norway). The results showed that the concentrations of Pb, Cd, Cu and Zn in the drinking water have been less than the standard level, and there is no concern for the public health. Dashtizadeh et al. [21] evaluate the concentrations of: As, Cd, Cr, Ni, Pb, B, Al, Hg, Mn, Zn, Cu, Fe, Se and Ba in tap water of Zahedan (Iran). A total of 155 samples of drinking water were randomly taken from the tap water and were analyzed using ICP-OES device. The hazard index (HI) values for children and adult age groups were 9.84E-01 and 4.22E-01, respectively. The cumulative Excess Lifetime Cancer Risk (ELCR) for carcinogenic trace elements was in range of tolerable carcinogenic risk 10^−6^ to 10^−4^. Another study from Iran, reported by Sarvestani and Aghasi [22], has been conducted to evaluate the concentrations of Pb, Cd and Cu in 160 drinking water samples in Kerman city, Iran. The results showed that the mean concentrations of lead metal in tap water have been higher than the recommended quantity based on the standards of the WHO and USEPA. In turn, 192 different drinking water sources in Chenzhou City of Hunan Province (China) were tested by Huang et al. [23]; carcinogenic and non-carcinogenic risk assessment was performed according to the method recommended by the US EPA. One more example from China concerns 116 tap water samples from Hunan province [24]; the results showed that 10% of the water samples exceeded the limit level of Cd established by WHO (0.003 mg L^−1^), 3% of the samples had Fe level and 1% had As level above the WHO limits (0.3 and 0.01 mg L^−1^), respectively. In the central area of Mexico, where the underground water was contaminated by natural origin arsenic and fluoride [25], in order to estimate health risk associated with human exposure to these pollutants, tap water samples from the southern-central region of the state were analyzed; 90% of the samples exceeded the levels of arsenic established by the WHO. The results of the conducted research are comparable with the data published by other authors cited in this work.

### Study limitation

Our research was limited to 18 metals and one year. A further limitation is that the interactions between different contaminants were not investigated as it would require a good knowledge of the mechanisms of toxicity for the different compounds. Recently, we are continuing them in a wider analytical scope (inorganic anions and cations as well as additional metals and metalloids), as well as sampling points (30 points, including water from other water suppliers).

## Conclusion

The wide range of concentration very probably origin from the erosion of sediment or corrosion of pipes and fittings used in the urban plumbing systems. The cumulative cancer risk of the target heavy metals for the children and adults using the tap water and bottled water are within the acceptable monitored and controlled levels (10^−4^ – 10^−6^). It was observed that the CDI values of non-carcinogenic metals were higher in children than in adults. The CDI values for adults and children were found in the order of: Zn > Sr > Cu > Mn > Ni > Pb > Cr > Co > As. The risks (HQ) caused by the non-carcinogenic metals decreased in the following order: Zn > Cu > Co > As > Sr > Pb > Cr > Ni > Mn. All the studied metals had HQ values below 1. Also HI values were less than 1. It means that the analyzed tap water is safe for human consumption. No significant seasonal changes in the concentrations of the tested metals were found. This proves the stability of the water and benefits its consumers. Although there is no potential health risk for children and adults, the children group consuming the tap water is at the risk of non-carcinogenic adverse health effect. Thus, it is recommended that the metals and metalloids concentrations should be periodically monitored more widely than official requirements to minimize the health risks in consumers.

## Data Availability

Not applicable.
